# Thrombospondin-1 Plays an Essential Role in Yes-Associated Protein Nuclear Translocation during the Early Phase of *Trypanosoma cruzi* Infection in Heart Endothelial Cells

**DOI:** 10.3390/ijms21144912

**Published:** 2020-07-12

**Authors:** Ashutosh Arun, Kayla J. Rayford, Ayorinde Cooley, Girish Rachakonda, Fernando Villalta, Siddharth Pratap, Maria F. Lima, Nader Sheibani, Pius N. Nde

**Affiliations:** 1 Department of Microbiology, Immunology and Physiology, Meharry Medical College, Nashville, TN 37208, USA; aarun@mmc.edu (A.A.); krayford@mmc.edu (K.J.R.); acooley@mmc.edu (A.C.); grachakonda@mmc.edu (G.R.); fvillalta@mmc.edu (F.V.); 2 School of Graduate Studies and Research, Meharry Medical College, Nashville, TN 37208, USA; spratap@mmc.edu (S.P.); mlima@med.cuny.edu (M.F.L.); 3 Department of Molecular Cellular and Biomedical Sciences, School of Medicine, The City College of New York, New York, NY 10031, USA; 4 Department of Ophthalmology and Visual Sciences, Biomedical Engineering and Cell and Regenerative Biology, University of Wisconsin School of Medicine and Public Health, Madison, WI 53705, USA; nsheibanikar@wisc.edu

**Keywords:** Chagas heart disease, heart endothelial cells, hippo signaling, transcriptional enhancer factor (TEF) family/TEA domain (TEAD) family, *Trypanosoma cruzi*, yes-associated protein1

## Abstract

The protozoan parasite *Trypanosoma cruzi* is the causative agent of Chagas disease. This neglected tropical disease causes severe morbidity and mortality in endemic regions. About 30% of *T. cruzi* infected individuals will present with cardiac complications. Invasive trypomastigotes released from infected cells can be carried in the vascular endothelial system to infect neighboring and distant cells. During the process of cellular infection, the parasite induces host cells, to increase the levels of host thrombospondin-1 (TSP-1), to facilitate the process of infection. TSP-1 plays important roles in the functioning of vascular cells, including vascular endothelial cells with important implications in cardiovascular health. Many signal transduction pathways, including the yes-associated protein 1 (YAP)/transcriptional coactivator, with PDZ-binding motif (TAZ) signaling, which are upstream of TSP-1, have been linked to the pathophysiology of heart damage. The molecular mechanisms by which *T. cruzi* signals, and eventually infects, heart endothelial cells remain unknown. To evaluate the importance of TSP-1 expression in heart endothelial cells during the process of *T. cruzi* infection, we exposed heart endothelial cells prepared from Wild Type and TSP-1 Knockout mouse to invasive *T*. *cruzi* trypomastigotes at multiple time points, and evaluated changes in the hippo signaling cascade using immunoblotting and immunofluorescence assays. We found that the parasite turned off the hippo signaling pathway in TSP-1KO heart endothelial cells. The levels of SAV1 and MOB1A increased to a maximum of 2.70 ± 0.23 and 5.74 ± 1.45-fold at 3 and 6 h, respectively, in TSP-1KO mouse heart endothelial cells (MHEC), compared to WT MHEC, following a parasite challenge. This was accompanied by a significant continuous increase in the nuclear translocation of downstream effector molecule YAP, to a maximum mean nuclear fluorescence intensity of 10.14 ± 0.40 at 6 h, compared to wild type cells. Furthermore, we found that increased nuclear translocated YAP significantly colocalized with the transcription co-activator molecule pan-TEAD, with a maximum Pearson’s correlation coefficient of 0.51 ± 0.06 at 6 h, compared to YAP-Pan-TEAD colocalization in the WT MHEC, which decreased significantly, with a minimum Pearson’s correlation coefficient of 0.30 ± 0.01 at 6 h. Our data indicate that, during the early phase of infection, upregulated TSP-1 is essential for the regulation of the hippo signaling pathway. These studies advance our understanding of the molecular interactions occurring between heart endothelial cells and *T. cruzi*, in the presence and absence of TSP-1, providing insights into processes linked to parasite dissemination and pathogenesis.

## 1. Introduction

*Trypanosoma cruzi,* the causative agent of Chagas disease, is an obligate intracellular hemoflagellate parasite that can infect all nucleated cells of the body. The disease, which was originally endemic in Mexico and other Latin American countries, is now present in all economically advanced countries of the world, including the United States, due to modern globalization making it a new global health threat [1,2,3,4,5]. Several modes of transmission including autochthonous transmission have been reported in the emerging infection regions of the world [6]. During the process of cellular infection, invasive *T. cruzi* trypomastigotes infected host cells and transform to replicative amastigotes within the infected cell. The amastigotes multiply and accumulate in the now distended cell, where they transform to invasive trypomastigotes, just before the cell burst, to release the invasive trypomastigotes. Some released trypomastigotes infect neighboring cells, while others are transported in blood through the host’s vascular system to infect cells in other parts of the body. During transportation in the vascular system, invasive trypomastigotes interact with, and potentially infect, endothelial cells lining the internal surface of the vascular wall.

Matricellular proteins are extracellular matrix (ECM) proteins that interact with cells and other ECM components to regulate cellular behavior and ECM organization, but are not part of the structural elements of the ECM [7,8,9,10]. Thrombospondin-1 (TSP-1) is a complex homotrimeric secreted matricellular glycoprotein, and a member of the group A subfamily of five TSP family members [11]. The protein plays important roles in regulating several cellular processes through its molecular interactions with extracellular molecules, including matrix regulating enzymes, glycosaminoglycans, growth factors and diverse cellular receptors among others, thereby having an important role in tissue and cellular homeostasis [9,12,13,14,15].

In the vasculature, TSP-1 plays an important role in the function of vascular cells, including vascular smooth muscle cells, endothelial cells, fibroblasts and inflammatory cells, suggesting that the molecule has important implications in cardiovascular health [16]. Furthermore, we showed that, during the early phase of infection, the parasite induces the expression of TSP-1 in host cells, including primary human coronary artery smooth muscle cells to facilitate cellular infection [17]. We also showed that the expressed TSP-1 interacts with *T. cruzi* calreticulin (TcCRT) on the surface of the parasite, to facilitate cellular infection, which was inhibited in the presence of the TcCRT monovalent Fab antibody [18]. Furthermore, an increase in the cellular expression of TSP-1 induced by the parasite is essential for dysregulating the levels of phosphorylated proteins and cellular signaling [19].

The Hippo signaling pathway, which was originally identified in *Drosophila melanogaster*, has now been extensively reported in mammalian cells. This pathway plays important evolutionarily conserved roles, in the regulation of organ size, cell proliferation, and apoptosis [20,21]. In mammals, when the canonical hippo pathway is turned on, the upstream mammalian sterile 20-like kinase 1/2 (MST1/2) is auto-phosphorylated, and it phosphorylates the regulatory protein SAV1. The complex phosphorylates the hydrophobic motifs of the large tumor suppressor kinase 1/2 (LATS1/2), which then phosphorylates the regulatory protein, MOB1. The phosphorylated and activated LATS1/2 then phosphorylate serine residues of hippo effector yes-associated protein (YAP), and the transcriptional coactivator with PDZ-binding motif (TAZ). The phosphorylation of YAP/TAZ is a signal for interaction with 14-3-3 for cytoplasmic retention or ubiquitination-mediated proteasomal and autolysosomal degradation [22,23,24]. Conversely, when the hippo pathway is turned off, MST1/2 are not phosphorylated, and they cannot signal the phosphorylation of downstream LATS1/2 and MOB1. Therefore, when YAP/TAZ are not phosphorylated, they can be translocated into the nucleus, where they interact with other TEA domain family members/transcription enhancer factor (TEADs/TEF) families of transcriptional co-activating factors, to promote the transcription of downstream genes, including TSP-1, which plays an important role in cell growth and proliferation [22]. Specifically, in a breast cancer model, it was suggested that, when the hippo signaling pathway is turned off, YAP-TEAD complex in the nuclear compartment directly induces the expression of TSP-1, which activates focal adhesion kinase, FAK [25]. The molecular mechanism by which *T. cruzi* signals and eventually infects endothelial cells remains to be elucidated. This knowledge will aid in the development of specific molecular intervention strategies.

We hypothesize that, during the early phase of molecular interaction between *T. cruzi* and heart endothelial cells, TSP-1 plays an important role in the dysregulation of the hippo signaling pathway. To delineate the significance of TSP-1 expression on hippo signaling during the early phase of infection, we challenged MHEC from wild type (WT) and TSP-1 knockout (TSP-1KO) mice with *T. cruzi*. We evaluated the kinetics of hippo signaling pathway, nuclear translocation of hippo effector molecule YAP, and its nuclear interaction with the co-activator protein TEAD. Here, we show that in the presence of TSP-1 (WT MHEC), the parasite induced a decrease in the unphosphorylated levels of SAV1 and MOB1A leading to a significant decrease in the nuclear translocation of YAP. This is accompanied by a decrease in nuclear co-localization of YAP and pan-TEAD. In the absence of TSP-1 (TSP-1 KO MHEC), the parasite induced an increase in the unphosphorylated levels of SAV1 and MOB1A, leading to an increase in the nuclear translocation of YAP. This increase in the nuclear translocation of YAP was accompanied by an increase in the nuclear co-localization of YAP and pan-TEAD.

## 2. Results

### 2.1. Kinetics of the Levels of SAV1 and MOB1A in MHEC during the Early Phase of T. cruzi Infection

**Figure 1:**
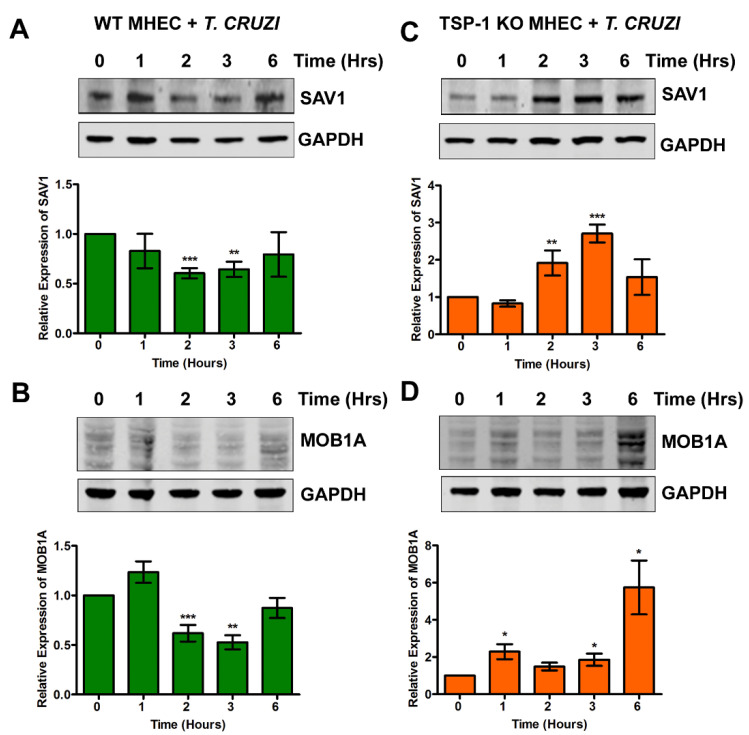
TSP-1 is essential for activation of hippo signaling cascade during the early phase of *T. cruzi* infection. Lysates (20 µg) from WT or TSP-1KO MHEC challenged with *T*. *cruzi* at different time points were resolved by SDS-PAGE, blotted, and probed with antibodies against (**A**) SAV1, (**B**) MOB1A in WT MHEC and (**C**) SAV1, (**D**) MOB1A in TSP-1KO MHEC, and developed as described. The blots were stripped, reprobed with antibodies against housekeeping GAPDH and developed with the corresponding IRDye conjugated secondary antibody. The developed blots were scanned using the infrared fluorescence detection Odyssey Imaging Systems. The normalized fold change in the level of each unphosphorylated protein was determined and plotted in the bar graph for WT MHEC (**A**, lower panel) SAV1, (**B**, lower panel) MOB1A, respectively and for TSP-1KO MHEC (**C**, lower panel) SAV1 and (**D**, lower panel) MOB1A, respectively. The bar graphs represent mean values ± SE from three independent biological replicates. The value of *p* < 0.05 was considered significant. * *p <* 0.05; ** *p <* 0.01; *** *p <* 0.001.

To gain insight into the importance of TSP-1 expression in the regulation of hippo signaling effector proteins and their associated signaling cascades in MHEC during the early phase of *T*. *cruzi* infection, we challenged WT and TSP-1 KO MHEC with *T*. *cruzi* for different lengths of time (0, 1, 2, 3 and 6 h), and analyzed the levels of unphosphorylated hippo signaling cascade proteins using immunoblot assays. We evaluated the levels of unphosphorylated SAV1 and MOB1A in WT and TSP-1KO MHEC at the different time points. We found that, when WT MHEC were challenged with *T. cruzi*, the levels of unphosphorylated SAV1 showed a general decrease trend that was very significant at 2 h (0.60 ± 0.05), *p* < 0.001 and at 3 h (0.64 ± 0.07)—*p* < 0.01 compared to uninfected control (Figure 1A).

This was accompanied by a significant decrease in the unphosphorylated levels of MOB1A, which is downstream of SAV1. The unphosphorylated levels of MOB1A increased at one hour then significantly decreased at 2 h (0.61 ± 0.08), *p* < 0.001 and at 3 h (0.52 ± 0.07), *p* < 0.01, compared to control (Figure 1B).

When we challenged TSP-1KO MHEC (in the absence of TSP-1), with the parasite, we observed that the levels of unphosphorylated SAV1 showed a continuous significant increase, from 2 h (1.91 ± 0.33), *p* < 0.01 to 3 h (2.70 ± 0.23), *p* < 0.001 before showing a non-significant increase at 6 h compared to control (Figure 1C). This was accompanied by an overall increase in the levels of unphosphorylated MOB1A, which was significantly increased at 1 h (2.28 ± 0.40), *p* < 0.05, 3 h (1.48 ± 0.21), *p* < 0.05, to a maximum of (5.74 ± 1.45), *p* < 0.05 at 6 h time point, compared to uninfected control (Figure 1D). These results indicate that the upstream Hippo signaling molecules are turned off in the absence of TSP-1 in MHEC challenged with *T. cruzi*.

### 2.2. Early T. cruzi Infection Increases Nuclear Translocation of YAP in TSP-1 KO MHEC

**Figure 2:**
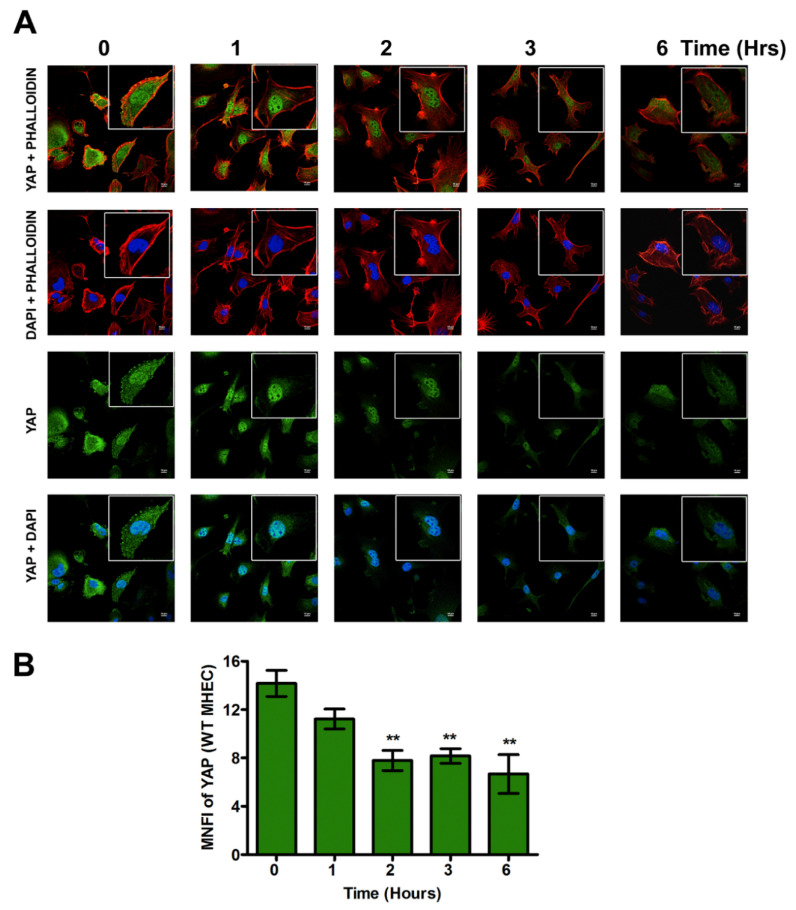
YAP is translocated into the nuclei of WT MHEC early during *T*. *cruzi* infection. WT MHEC grown on coverslips coated with 1% gelatin in 6-well culture plates were challenged with *T*. *cruzi* at different time points, washed, fixed, perforated with 0.1% Triton-X100, blocked with 3% BSA-PBS, and incubated at 4 °C overnight in solutions containing phalloidin and (**A**) mouse anti YAP antibodies. The cells were washed and reprobed with goat anti mouse Alexa Fluor 488 conjugated secondary antibody. The washed cells were mounted with mounting media containing DAPI to stain the nuclei. (**B**) Stained slides were analyzed by confocal microscopy and the mean nuclear fluorescence intensity (MNFI) values were plotted for YAP. Images were captured at 60× at scale bar 10 μm. Each confocal microscopy image is a representative of three independent biological replicates. The bar graphs represent MNFI values ± SE from three independent biological replicates. The value of *p <* 0.05 was considered significant. ** *p <* 0.01.

Our data suggest that *T. cruzi* turns off the Hippo signaling pathway in the absence of TSP-1. When the hippo signaling is turned off, the downstream effector molecules are not phosphorylated, and therefore can be translocated into the nucleus. To evaluate if the increase in the levels of unphosphorylated upstream hippo signaling molecules correspond with an increase in nuclear translocation of downstream hippo effector molecule YAP, we challenged WT or TSP-1 KO MHEC with *T*. *cruzi* at different time points (0, 1, 2, 3 and 6 h), and analyzed the levels of nuclear translocation of downstream hippo effector molecule YAP, using confocal microscopy assays. We observed that when WT MHEC were challenged with *T. cruzi* trypomastigotes, the level of nuclear translocation of YAP significantly decreased with time, as shown by the nuclear intensity of the fluorophore in several fields. The mean nuclear intensity of YAP bound to the primary antibody detected by Alexa Fluor 488 conjugated secondary antibody decreased from 1 h, and the value became significant from 2 h (7.78 ± 0.83), *p* < 0.01, and continued to 3 h (8.16 ± 0.01), *p* < 0.01 and finally 6 h (6.7 ± 1.60), *p* < 0.01 compared with control (Figure 2A,B).

**Figure 3:**
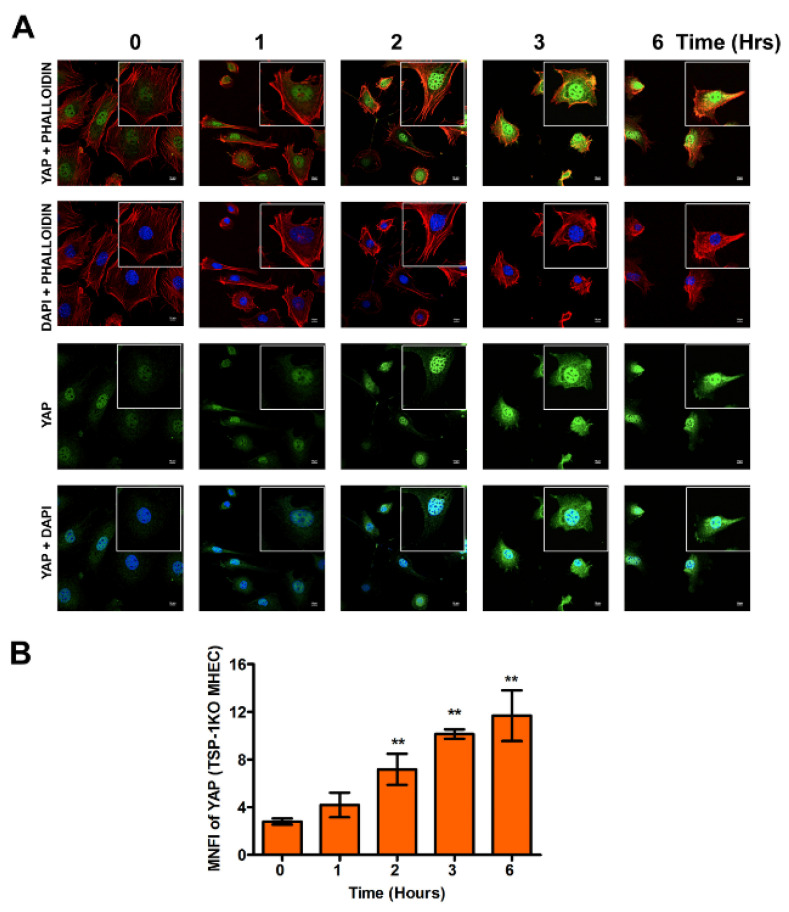
*T. cruzi* induces significant translocation of YAP into the nuclei of TSP-1KO MHEC early during *T*. *cruzi* infection. TSP-1KO MHEC grown on coverslips coated with 1% gelatin in 6 well culture plates were challenged with *T*. *cruzi* at different time points, washed, fixed, perforated with 0.1% Triton-X100, blocked with 3% BSA-PBS, and incubated at 4 °C overnight, in solutions containing phalloidin and (**A**) mouse anti YAP antibodies. The cells were washed and reprobed with goat anti mouse Alexa Fluor 488 conjugated secondary antibody. The washed cells were mounted with mounting media containing DAPI to stain the nuclei. (**B**) Stained slides were analyzed by confocal microscopy and the mean nuclear fluorescence intensity (MNFI) values were plotted for YAP. Images were captured at 60× at scale bar 10 μm. Each confocal microscopy image is a representative of three independent biological replicates. The bar graphs represent MNFI values ± SE from three independent biological replicates. The value of *p <* 0.05 was considered to be significant. ** *p <* 0.01.

Conversely, when we challenged TSP-1KO MHEC with the parasite, we observed that the nuclear intensity of YAP translocated into the nucleus showed a gradual continuous significant increase from 2 h (7.17 ± 1.30), *p* < 0.01 to 3 h (10.14 ± 0.40), *p* < 0.01, and finally to a maximum at 6 h (11.68 ± 2.12), *p* < 0.01 compared to control (Figure 3A,B).

A comparison of the differences in the nuclear translocation of YAP showed that in the presence of TSP-1, there is a significant continuous decrease in YAP nuclear translocation with time, compared to the absence of TSP-1 where we observe a significance continuous increase in YAP nuclear intensity. At 6 h post infection, WT MHEC had a higher number of parasites per cell (4.2 ± 0.15) than TSP-1 KO MHEC (0.12 ± 0.02).

### 2.3. YAP and Pan-TEAD Co-Localize in the Nucleus of MHEC during the Early Phase of T. cruzi Infection

**Figure 4:**
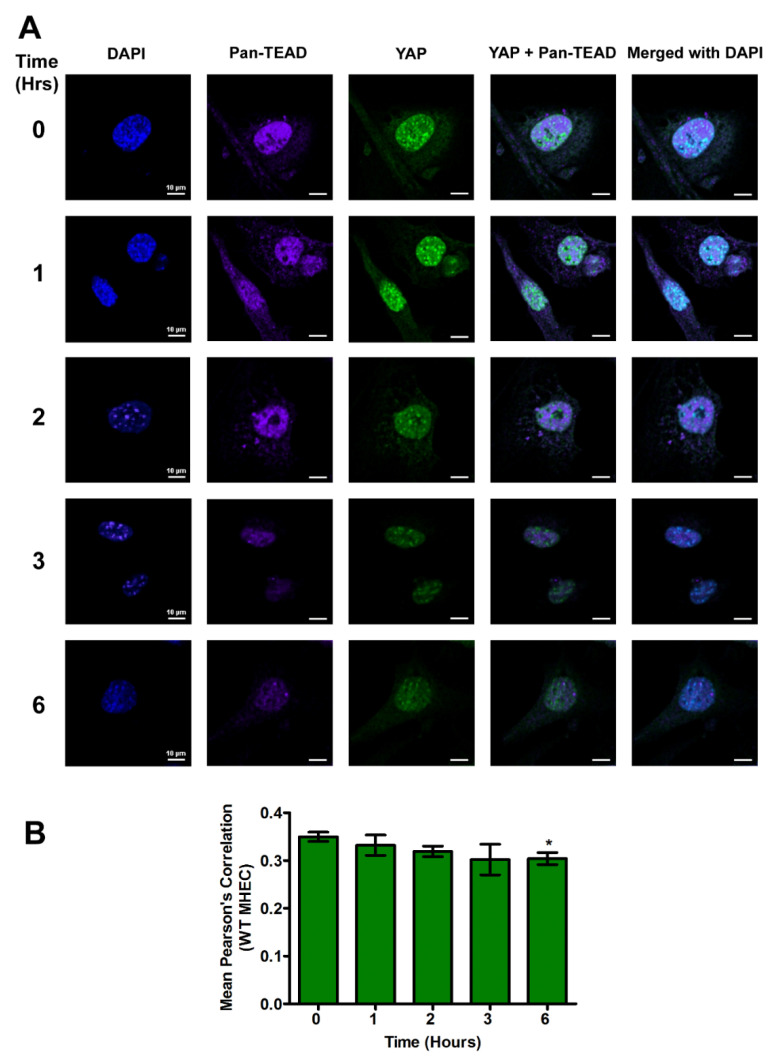
YAP and pan-TEAD are colocalized in the nuclei of WT MHEC during the early phase *T. cruzi* infection. WT MHEC grown on coverslips coated with 1% gelatin in 6 well culture plates were challenged with *T. cruzi* at different time points, washed, fixed, perforated with 0.1% Triton-X100, blocked with 3% BSA-PBS, and incubated at 4 °C overnight, in solutions containing phalloidin and (**A**) mouse anti YAP and rabbit anti pan-TEAD antibodies. The washed cells were reprobed with a cocktail of goat anti mouse Alexa Fluor 488 and goat anti rabbit Alexa Fluor 647 conjugated secondary antibodies. The cells were washed and mounted with mounting media containing DAPI to stain the nuclei. Stained slides were analyzed by confocal microscopy and images were captured at 60× at scale bar 10 μm. The mean fluorescence intensities of the merged signals were analyzed using confocal microscopy software to generate Pearson’s correlation coefficients. (**B**) The bar graphs represent Pearson’s correlation coefficients values ± SE from three independent biological replicates. Each confocal microscopy image is a representative of three independent biological replicates. The value of *p* < 0.05 was considered significant. * *p* < 0.05.

The downstream hippo effector molecule YAP is a transcription factor that interacts with its co-activator TEAD to bind to DNA. To evaluate whether the YAP translocated to the nucleus interacts with TEAD, MHEC (WT and TSP-1 KO) were challenged with *T. cruzi* (10 parasites per cell), then the cells were harvested or fixed after different time points after infection (0, 1, 2, 3 and 6 h). Then, they were analyzed for the levels of nuclear co-localization of the downstream effector molecule YAP with TEAD, by measuring the Pearson’s correlation of both proteins in the nucleus using confocal microscopy assays. In the presence of TSP-1 in WT MHEC, we observed that the mean Pearson’s correlation of YAP and pan-TEAD nuclear colocalization decreased gradually with time, and was significant at 6 h (0.30 ± 0.01), *p* < 0.05, compared to control (Figure 4A,B).

**Figure 5:**
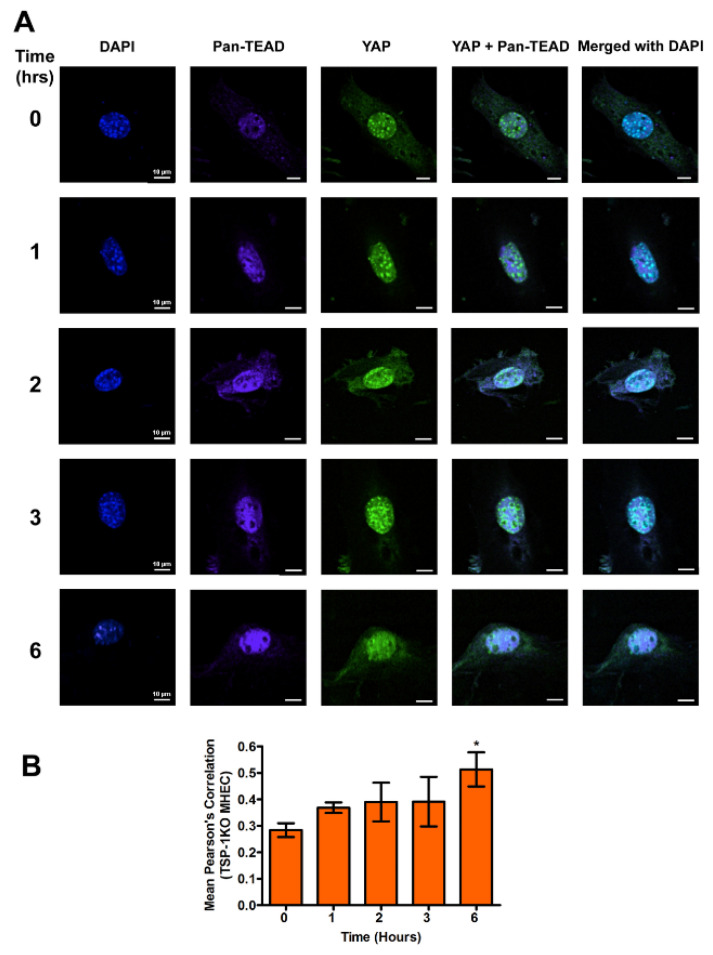
Transcriptional co-activators YAP and pan-TEAD are colocalized in the nuclei of TSP-1KO MHEC during the early phase of *T. cruzi* infection. TSP-1KO MHEC grown on coverslips coated with 1% gelatin in 6 well culture plates were challenged with *T. cruzi* at different time points, washed, fixed, perforated with 0.1% Triton-X100, blocked with 3% BSA-PBS, and incubated at 4 °C overnight in solutions containing phalloidin, and the following antibodies; (**A**) mouse anti YAP and rabbit anti pan-TEAD antibodies. The cells were washed and reprobed with a cocktail of goat anti mouse Alexa Fluor 488 and goat anti rabbit Alexa Fluor 647 conjugated secondary antibodies. The cells were washed and mounted with mounting media containing DAPI to stain the nuclei. Stained slides were analyzed by confocal microscopy and images were captured at 60× at scale bar 10 μm. Each confocal microscopy image is a representative of three independent biological replicates. The mean fluorescence intensities of the merged signals were analyzed using confocal microscopy software to generate Pearson’s correlation coefficients. (**B**) The bar graphs represent Pearson’s correlation coefficients values ± SE from three independent biological replicates. The value of *p* < 0.05 was considered to be significant. * *p* < 0.05.

In the absence of TSP-1, we observed a gradual increase in the nuclear colocalization of YAP and pan-TEAD, as shown by a similar increase in mean Pearson’s correlation coefficient to a maximum at 6 h (0.51 ± 0.06), *p* < 0.05, compared to uninfected control (Figure 5A,B).

A comparison of the nuclear colocalization of YAP and pan-TEAD showed a gradual decrease in WT MHEC compared to a gradual increase in the absence of TSP-1 in MHEC TSP-1KO, all of which were significant at 6 h time point, compared to control.

## 3. Discussion

Chagas disease is a major pathology associated with severe morbidity and mortality in *T*. *cruzi*-infected patients. The molecular mechanisms through which the parasite causes heart disease remains to be clearly elucidated despite ongoing research. Cardiac pathology caused by *T. cruzi* infection includes the general cardiac enlargement of all four chambers—myocarditis, vasculitis, and vascular dilation, among others [26]. The parasite can infect all nucleated cells of the heart, including myocytes, fibroblasts, neurons, adipocytes and endothelial cells [2,27,28]. Invasive trypomastigotes carried in the blood will interact with and infect heart endothelial cells causing disease. However, the molecular mechanisms involved remain undefined.

We showed previously that the parasite upregulates host TSP-1 during the early phase of infection and the knock down of host TSP-1 by RNAi reduces the level of cellular infection by *T. cruzi* [17]. Furthermore, we also showed that the parasite interacts with host TSP-1 through *T. cruzi* calreticulin (TcCRT) located on the surface of the parasite [18]. The preincubation of host cells with an inhibitor of janus kinase (JNK) which is upstream of TSP-1, reduced the upregulation of TSP-1 induced by the parasite during the early phase of infection, indicating that TSP-1 upregulation is important and plays an essential role during infection [19]. The host cell signaling mechanism(s) regulated by the expressed host TSP-1 to facilitate infection and *T. cruzi* mediated pathogenesis remains to be elucidated. Elucidating the roles that host TSP-1 plays during the early phase of cellular infection, including the dysregulation of host cell signaling responses, will enhance our understanding of *T. cruzi* pathogenesis.

Although TSP-1 is one of the downstream genes of the hippo signaling pathway, which has been reported to play a role in organ enlargement and fibrogenic responses, the role that it may play in the regulation of hippo signaling pathway in heart endothelial cells during the early phase of *T. cruzi* infection is unknown [14,20,21,25]. Therefore, we hypothesize that *T. cruzi* trypomastigotes will dysregulate the hippo signaling pathway in heart endothelial cells during the early phase of cellular infection in a TSP-1 dependent manner. To elucidate the significance of TSP-1 in hippo signaling during the early phase of cellular infection of heart endothelial cells, we challenged MHEC from WT and TSP-1KO mice with invasive *T*. *cruzi* trypomastigotes, for various lengths of time (0, 1, 2, 3 and 6 h), and evaluated whether the hippo signaling pathway was turned on or off in the presence or absence of TSP-1. Our results show that the parasite turned “off” the hippo signaling pathway of MHEC in a TSP-1 dependent manner. Specifically, in the absence of TSP-1, the levels of unphosphorylated SAV1 and MOB1A proteins, which play an important role in hippo signaling [29,30], were significantly increased (Figure 1C,D). Our findings agree with others who suggested that the levels of unphosphorylated YAP is increased when SAV1 and MOB1A are not phosphorylated [31]. In the absence of TSP-1 in TSP-1 KO MHEC, the level of YAP significantly increased with time. The parasite turned “on” the hippo signaling pathway cascade proteins of WT MHEC, which expresses TSP-1 (Figure 1A,B). Furthermore, we showed that the levels of the transcriptional co-activator, YAP, translocated in to the nucleus significantly increased with time to a maximum at 6 h in the TSP-1 KO MHEC (Figure 3A,B). Our data agree with reports in the literature, suggesting that when the hippo signaling pathway is turned off, the downstream effector transcriptional co-activator molecule YAP is translocated into the nucleus [32,33]. Meanwhile, in the presence of TSP-1, as in WT MHEC, the hippo signaling pathway is turned on, and the levels of transcriptional co-activator translocated to the nucleus significantly decrease with time (Figure 2A,B). Furthermore, in agreement with the upstream observations, our data showed that nuclear translocated YAP colocalized with its transcriptional co-activator TEAD. An increase in the levels of nuclear translocated YAP leads to an increase in its nuclear colocalization with pan-TEAD, in the absence of TSP-1 (Figure 5A,B), compared to a decrease in the nuclear YAP-pan-TEAD colocalization in WT MHEC that expresses TSP-1 (Figure 4A,B). Our observations support the current literature, which suggests that YAP interacts with its transcriptional co-activator pan-TEAD in the nuclear compartment. These results show, for the first time, that *T*. *cruzi* infection enhances nuclear translocation of YAP and the colocalization of YAP with pan-TEAD in the nuclei of TSP-1 KO MHEC compared to WT MHEC.

We showed that *T. cruzi* upregulates the levels of TSP-1 during the process of infection [19,34]. Others showed that TSP-1 is one of the downstream molecules of the hippo signaling cascade [22,25]. This suggests that, in the absence of TSP-1, the parasites turned off the hippo signaling pathway, in an attempt to increase the level of host TSP-1, which is important in infection. Additionally, it was suggested that the level of TGF-β increases during the process of *T. cruzi* infection, and TGF-β inhibitor therapeutic use decreased fibrogenic responses during *T. cruzi* infection [35,36,37]. Others have suggested that TSP-1 can activate TGF-β, a profibrotic cytokine [38,39,40]. Taken together, this further supports our concept that an increase in the level of TSP-1 induced by the parasite is essential for *T. cruzi* infection and pathogenesis.

## 4. Materials and Methods

### 4.1. Ethics Statement

The animal study was carried out in accordance with the protocol number 150514PN093, approved by the Institutional Animal Care and Use Committee (IACUC) of Meharry Medical College.

### 4.2. Generation and Culture of Mouse Heart Endothelia Cells (MHEC)

MHEC were generated and maintained, as previously described for retinal EC [41]. Briefly, hearts were harvested from 4-week-old wild type or TSP-1KO Immorto mice, as detailed [41]. Hearts (from 3–4 mice) were pooled together, rinsed with plane DMEM (non-supplemented), minced into small pieces in a 60 mm tissue culture dish using sterilized razor blades, and digested in 5 mL of collagenase type I (1 mg/mL in serum free DMEM, Worthington, Lakewood, NJ, USA), for 30–45 min at 37 °C. Following digestion, DMEM with 10% FBS was added, and cells were pelleted. The cellular digests were then filtered through a double layer of sterile 40 μm nylon mesh (Sefar America Inc., Fisher Scientific, Hanover Park, IL, USA), and centrifuged at 400× *g* for 10 min to pellet cells. Cells were washed twice with DMEM containing 10% FBS, resuspended in 1.5 mL medium (DMEM with 10% FBS), and incubated with sheep anti-rat magnetic beads, pre-coated with anti-PECAM-1 as described [41]. After affinity binding, magnetic beads were washed six times with DMEM with 10% FBS and bound cells in endothelial cell growth medium were plated into a single well of a 24 well plate, pre-coated with 2 μg/mL of human fibronectin (BD Biosciences, Bedford, MA, USA). Endothelial cells were grown in DMEM containing 10% FBS, 2 mM l-glutamine, 2 mM sodium pyruvate, 20 mM HEPES, 1% non-essential amino acids, 100 μg/mL streptomycin, 100 U/mL penicillin, freshly added heparin at 55 U/mL (Sigma, St. Louis, MO, USA), endothelial growth supplement 100 μg/mL (Sigma, St. Louis, MO, USA), and murine recombinant interferon-γ (R & D, Minneapolis, MN, USA), at 44 units/mL. Cells were maintained at 33 °C with 5% CO_2_. Cells were progressively passed to larger plates, maintained, and propagated in 1% gelatin-coated 60 mm dishes. To confirm that these cells are EC, we examined the expression of two endothelial cell specific markers, PECAM-1 and VE-cadherin, by FACS analysis. Nearly 100% of wild type and TSP1^−/−^ MHEC expressed high levels of these markers on their surface (not shown). Early passage cultures of MHEC wild type (WT MHEC) and TSP-1KO (TSP-1KO MHEC) were maintained in growth medium and used for the studies outlined here.

### 4.3. Rat Heart Myoblast Culture

Rat heart myoblasts (RHM) were maintained in DMEM containing glutamax (Life Technologies, Grand Island, NY, USA), 10% heat inactivated fetal bovine serum (Life Technologies), 1% penicillin/streptomycin (Life Technologies) and 1% non-essential amino acid and multivitamin (Life Technologies). RHM were grown in a humidified tissue culture incubator at 37 °C and 5% CO_2_.

### 4.4. T. cruzi Trypomastigote Culture and Infection Assays

RHM monolayers were cultured in complete media to 80% confluence. The cells were infected with *T. cruzi* trypomastigotes clone MMC 20A (Tulahuen strain). Infected heart myoblast monolayers were fed with fresh complete media (supplemented media) daily. A pure population of highly invasive *T. cruzi* trypomastigotes released in cell culture supernatants were harvested as previously described [42,43,44]. The parasites were washed in Hanks’ balanced salt solution (HBSS) and resuspended in MHEC growth medium without supplement, at a working concentration of 1 × 10^7^ parasites/mL, for use in subsequent experiments. For infection assays, approximately 85% confluent MHEC (WT or TSP-1KO) monolayers were starved in medium without supplements, followed by the addition of invasive *T. cruzi* trypomastigotes, at a ratio of 10 parasites per cell. The cells challenged with the parasites were incubated for different time points—1, 2, 3 and 6 h. Parasites were washed off with 1 × DPBS (without calcium/magnesium), and the cells were either processed immediately, or stored at −80 °C for western blot analysis. Each time point was done using three independent T75 flask, for use in the western blotting experiments, and three independent sets of 6 well culture plates containing coverslip coated with 1% gelatin, for use in confocal immunofluorescence microscopy assays. Mock-infected (medium only) MHECs were used as controls for each time point.

### 4.5. Immunoblotting Assays

Uninfected control and parasite challenged cell monolayers were lysed in RIPA buffer (Life Technologies), containing protease inhibitor cocktail set III at 1:100, (Calbiochem, Gibbstown, NJ, USA) and phosphatase inhibitor cocktails 2 and 3, at 1:100 each (Sigma Aldrich, St. Louis, MO, USA). Whole cell lysates (20 μg/well) were separated by SDS-PAGE on a 4–15% gradient polyacrylamide gels, and transferred onto nitrocellulose membranes (Life Technologies). The membranes were incubated in Intercept TBS Blocking Buffer (LI-COR Biosciences, Lincoln, Nebraska, USA), followed by incubation with an appropriate primary antibody diluted at 1:1000 at 4 °C overnight on a shaker; mouse anti-SAV1 monoclonal antibody (Santa Cruz Biotechnology, Dallas, TX, USA; cat. no. sc-374366), mouse anti-GAPDH monoclonal antibody (Santa Cruz Biotechnology, cat. no. sc-47724), mouse anti-MOB1A monoclonal antibody (Life Technologies, catalog. no. MA5-31801). The blots were washed and incubated with the corresponding IRDye secondary antibody (LI-COR 800CW or 680RD anti-mouse/anti-rabbit) in blocking buffer containing 0.01% Tween 20 for 1 h at room temperature. The blots were washed and scanned using the infrared fluorescence detection Odyssey Imaging Systems (LI-COR Biosciences) to visualize the bound antibody. Housekeeping GAPDH signal was used for normalization of loading differences. Each experiment was done in triplicate, and the quantitation of band intensity was performed by densitometry using Image J. A statistical analysis of each parameter for the *T. cruzi* treated groups was compared with non-treated control groups (0 Hours) using student’s *t*-test or one-way ANOVA (non-parametric), with Newman–Keuls post-hoc test. The difference was considered statistically significant if * *p* < 0.05, ** *p* < 0.01 and *** *p* < 0.001 vs. control.

### 4.6. Immunofluorescence Assays

MHECs (WT or TSP-1KO) were seeded on coverslip coated with 1% gelatin in 6 well culture plate. *T*. *cruzi* trypomastigotes Tulahuen strain clone MMC 20A (10 parasites per cell) were incubated with the MHEC for different time points—1, 2, 3 and 6 h. The parasites were washed off with DPBS. The cells were fixed with 4% paraformaldehyde for 5 min at room temperature and washed with 1X DPBS. Fixed cells were perforated with 0.1% Triton-X100 in TBS for 5 min, and blocked with 3% BSA-PBS for 30 min at room temperature. For an evaluation of nuclear translocation, slides were incubated with mouse anti-YAP monoclonal antibody diluted 1:100 (Santa Cruz Biotechnology, cat. no. sc-271134) and phalloidin (1:2000) in 1% BSA-PBS at 4 °C overnight. The cells were washed with 1% BSA-PBS and re-probed with goat anti mouse IgG secondary antibody conjugated with Alexa Fluor 488 (1:1000) in wash buffer for 1 h. They were further washed and mounted with mounting media containing DAPI (Life Technologies) to stain the nuclei. For colocalization assays, the fixed, perforated and blocked cells on the slides were incubated with a mixture of antibodies, containing mouse anti-YAP monoclonal antibody diluted 1:100 (Santa Cruz Biotechnology cat no. sc-271134), rabbit anti-pan-TEAD (D3F7L) monoclonal antibody (Cell signaling Technology, cat no.13295) diluted 1:100, and phalloidin (1:2000) at 40 °C overnight. The cells were washed and the bound primary antibody were detected using the corresponding secondary antibody cocktails; goat anti-rabbit Alexa Fluor 647 and goat anti-mouse Alexa Fluor 488 antibodies, diluted 1:1000 in 1% BSA-PBS, for 1 h at room temperature, washed and mounted with mounting media containing DAPI to stain the nuclei. The coverslips with treated cells were transferred on to glass slides and sealed. Stained slides were analyzed using the Nikon A1R confocal microscope, located at the Centralized Core Facility at Meharry Medical College. Cellular and nuclear intensities, including Pearson’s correlation coefficient for the colocalization of signals were determined by imaging software NIS Elements AR Analysis version 5.20.02 64-bit.

### 4.7. Statistical Analysis

Data from at least three independent experiments are expressed as mean ± SEM. Statistical comparisons were made between controls and *T. cruzi* treated groups. Statistical comparisons were analyzed using Student’s *t*-test or a one-way analysis of variance (ANOVA) for multiple groups of data, followed by a Newman–Keuls test. *p*-value strength increases with number of asterisks *p* < 0.05 (*), *p* < 0.01 (**), and *p* < 0.001 (***). Statistical analyses were done using GraphPad Prism (GraphPad Software, San Diego, CA, USA).

## Abbreviations

DMEM	Dulbecco’s modified eagle medium
FBS	Fetal bovine serum
MHEC	Mouse heart endothelia cells
SAV1	Salvador homolog 1
TAZ	Transcriptional coactivator with PDZ-binding motif
TcCRT	*Trypanosoma cruzi* calreticulin
TEAD	Transcriptional enhancer factor (TEF) family/TEA domain (TEAD) family
TSP-1KO	Thrombospondin-1 knock out
WT	Wild type
YAP	Yes-associated protein
